# Antimicrobial efficacy and toxicity of novel CAMPs against *P. aeruginosa* infection in a murine skin wound infection model

**DOI:** 10.1186/s12866-019-1657-6

**Published:** 2019-12-16

**Authors:** Ming Yang, Chunye Zhang, Sarah A. Hansen, William J. Mitchell, Michael Z. Zhang, Shuping Zhang

**Affiliations:** 1 Department of Veterinary Pathobiology, College of Veterinary Medicine, University of Missouri, Columbia, MO 65211 USA; 2 Office of Animal Resources, University of Missouri, Columbia, MO 65211 USA; 3 Veterinary Medical Diagnostic Laboratory, College of Veterinary Medicine, University of Missouri, Columbia, MO 65211 USA; 4 Department of Biomedical Science, College of Veterinary Medicine, University of Missouri, Columbia, MO 65211 USA

**Keywords:** Cationic antimicrobial peptides, Toxicity, Antimicrobial activity, *Pseudomonas aeruginosa*, Mouse model, Skin infection

## Abstract

**Background:**

Treatment of *P. aeruginosa* wound infection is challenging due to its inherent and acquired resistance to many conventional antibiotics. Cationic antimicrobial peptides (CAMPs) with distinct modes of antimicrobial action have been considered as the next-generation therapeutic agents. In the present study, a murine skin surgical wound infection model was used to evaluate the in vivo toxicity and efficacy of two newly designed antimicrobial peptides (CAMP-A and CAMP-B), as chemotherapeutic agents to combat *P. aeruginosa* infection.

**Results:**

In the first trial, topical application of CAMPs on the wounds at a dose equivalent to 4 × MIC for 7 consecutive days did not cause any significant changes in the physical activities, hematologic and plasma biochemical parameters, or histology of systemic organs of the treated mice. Daily treatment of infected wounds with CAMP-A and CAMP-B for 5 days at a dose equivalent to 2× MIC resulted in a significant reduction in wound bacterial burden (CAMP-A: 4.3 log_10_CFU/g of tissue and CAMP-B: 5.8 log_10_CFU/g of tissue), compared to that of the mock-treated group (8.1 log_10_CFU/g of tissue). Treatment with CAMPs significantly promoted wound closure and induced epidermal cell proliferation. Topical application of CAMP-A on wounds completely prevented systemic dissemination of *P. aeruginosa* while CAMP-B blocked systemic infection in 67% of mice and delayed the onset of systemic infection by at least 2 days in the rest of the mice (33%). In a second trial, daily application of CAMP-A at higher doses (5× MIC and 50× MIC) didn’t show any significant toxic effect on mice and the treatments with CAMP-A further reduced wound bacterial burden (5× MIC: 4.5 log_10_CFU/g of tissue and 50× MIC: 3.8 log_10_CFU/g of tissue).

**Conclusions:**

The data collectively indicated that CAMPs significantly reduced wound bacterial load, promoted wound healing, and prevented hepatic dissemination. CAMP-A is a promising alternative to commonly used antibiotics to treat *P. aeruginosa* skin infection.

## Background

*P. aeruginosa* is a leading cause of opportunistic infections including wounds, respiratory system and eye infections [1]. On an annual basis, approximately 13% of 51,000 cases of *P. aeruginosa* infections is involved in multidrug-resistant strains in the United States, resulting in more than 400 deaths (http://www.cdc.gov/hai/organisms/pseudomonas.html). *P. aeruginosa* colonization of wounds and its rapid proliferation within the damaged tissues often leads to systemic dissemination [2]. One of the contributing factors to the poor prognosis of *P. aeruginosa* infection is the organism’s inherent resistance to many commonly used antibiotics [3, 4]. Currently, beta-lactam antibiotics (e. g. imipenem) alone, quinolones (e.g. ciprofloxacin) alone, or in combination with aminoglycosides (e. g. gentamicin) are the primary therapeutic agents against *P. aeruginosa* infections. However, the increase in resistance rates to beta-lactams, aminoglycosides, and quinolones [5, 6] reduces the treatment options [7].

Recently, host cationic antimicrobial peptides (CAMPs) constituting the first line of innate defense [8] have been explored as a new class of antimicrobial agents [9, 10]. These peptides kill microbial pathogens via nonspecific membrane-disruption and binding to intracellular components, which makes it more difficult for microbes to develop resistance [11]. To date, approximately 10 peptides are under clinical trials and several others are at the preclinical stage (mainly for topical use) [12]. Avian β-defensins (AvBDs) are host antimicrobial peptides that have pleiotropic biological functions, such as broad-spectrum antimicrobial activity, LPS-neutralizing ability, and chemotactic activity [13–16]. A series of structurally simple AvBD analogues with increased antimicrobial activity have been designed and served as templates for further optimizations [15]. Two short α-helical peptides (CAMP-A and CAMP-B) consisting of key domains and residues of AvBD-12 and AvBD-6 demonstrated potent antimicrobial activity, had resistance to salts and proteases, and minimal cytotoxicity to host cells. In vitro study indicated that CAMP-A and CAMP-B effectively inhibited the growth of *P. aeruginosa*, *Staphylococcus aureus*, and *Staphylococcus pseudintermedius,* including clinical isolates resistant to β-lactam antibiotics, chloramphenicol, tetracycline, and sulfamethoxazole [17]. The objective of this study was to evaluate the efficacy of CAMP-A and CAMP-B against *P. aeruginosa* using a murine skin surgical wound infection model.

## Results

### Verification of *P. aeruginosa* infection in mice

**Fig. 1:**
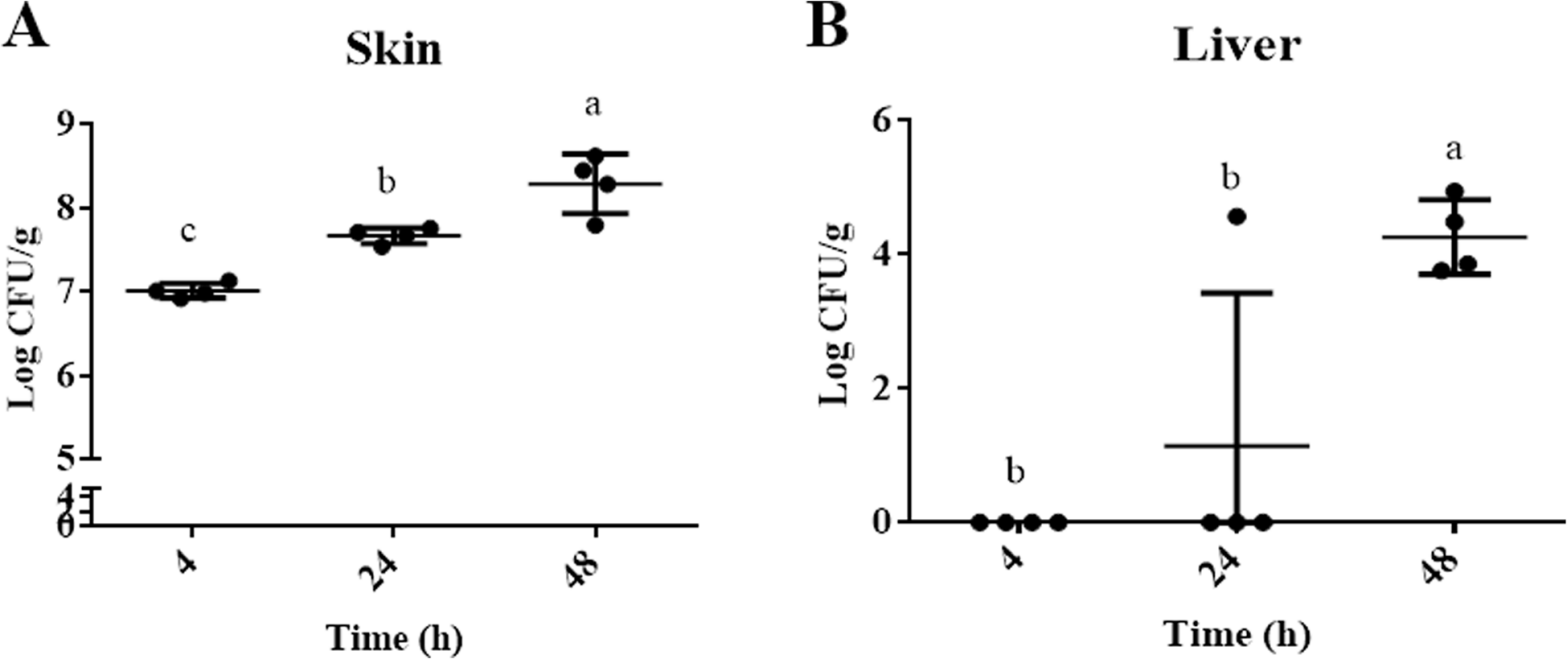
Establishment of *P. aeruginosa* wound infection. Bacteria (2.5 × 10^6^ CFU) were inoculated into each skin surgical wound. The inoculated mice were sacrificed at 4, 24, and 48 h post-infection. Serial dilutions of tissue homogenates were cultured on LB agar and incubated at 37 °C overnight followed by enumeration of bacterial colony-forming units (CFU). **a**_Log10_ CFU/g of wounded skin tissue. **b** Log_10_ CFU/g of liver tissue. Data are presented as average ± standard deviations (SD, *n* = 4). Different lowercase letters (a, b, c) above the bars show significant differences (*p* < 0.05) among different time points. Each letter represents a level of significant difference

Following inoculation of the wound with *P. aeruginosa*, the average CFU burden in the wounded skin was 7.0 log_10_ CFU/g at 4 h, 7.67 log_10_ CFU/g at 24 h, and 8.29 log_10_ CFU/g at 48 h (Fig. 1a), respectively. *P. aeruginosa* was first detected in the liver of one out of four mice at 24 h (Fig. 1b). At 48 h post-inoculation, the bacteria spread to the liver of all mice and the average CFU counts were around 4 log10 CFU/g (Fig. 1b). The recovered bacteria were confirmed to be *P. aeruginosa* using a matrix-assisted laser desorption/ionization-time of flight mass spectrometry (MALDI-TOF-MS) [18].

### Toxicity of CAMP-A and CAMP-B in mice

**Fig. 2:**
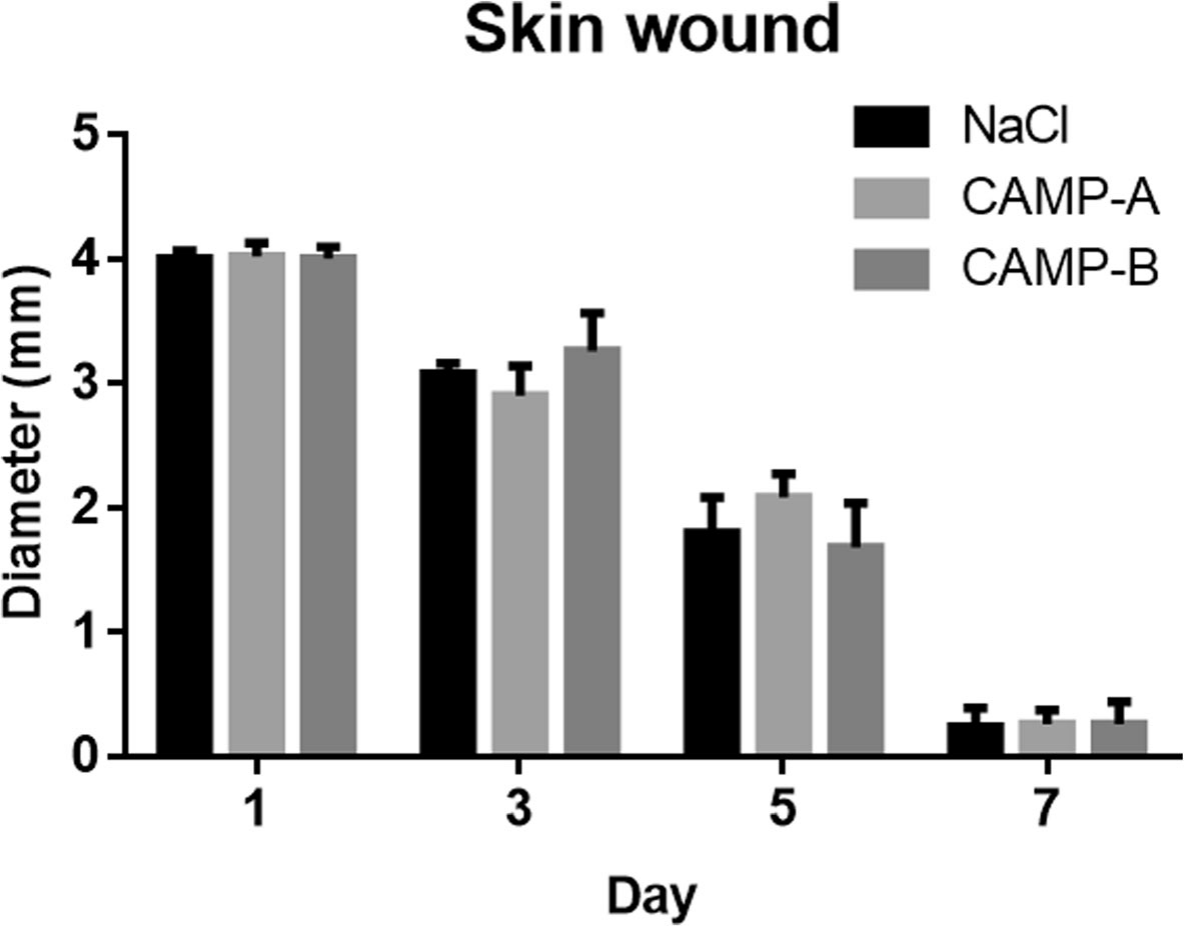
The effect of CAMPs on skin wound healing of the treated mice. Following administration of peptides (4 × MIC) or 0.9% NaCl, the wound diameter was measured at day 1, 3, 5, and 7. Data are presented as means ± SD (*n* = 6). No significant difference was observed among treatment groups at any given time point

**Fig. 3:**
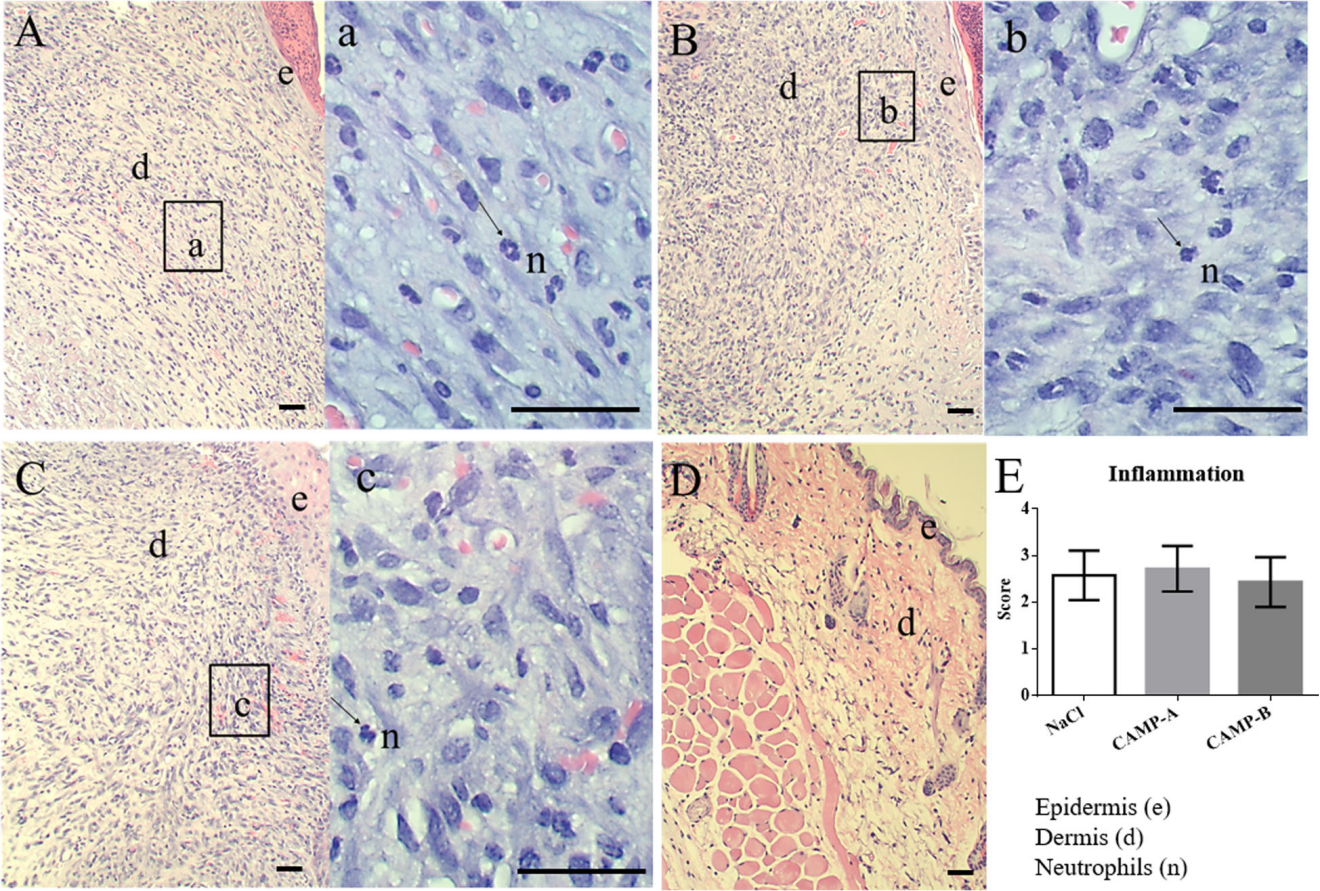
Toxic effect of CAMPs on the histology of the wounded skin. The wounds were treated daily with CAMP-A, CAMP-B and 0.9% NaCl. On day 7, wounded skin tissues were excised and fixed in 10% of formalin, embedded in paraffin, sectioned at 5-μm thickness, and stained with hematoxylin and eosin (H&E). **a** 0.9% NaCl, (**b**) 4 × MIC CAMP-A, (**c**) 4 × MIC CAMP-B, and (**d**) Skin prior to wound creation. Bar = 100 μm. Representative areas (rectangle) are shown as enlarged images. **e** Scores of the inflammatory responses in the three treatment groups. The inflammatory infiltration consisted of lymphocytes, macrophages, and neutrophils. Letters indicate the following cell types: e, epidermis; d, dermis; n, neutrophils. Data are presented as means ± SD (*n* = 6)

In the first toxicity study, no significant differences in body weight, behavior, and hair coat were observed among the treatment groups after daily application of 4 × MIC CAMPs, and NaCl for 7 days (Additional file 1: Figure S1). In addition, the closure of skin wound was similar among the treatment groups, including 0.9% NaCl, CAMP-A, and CAMP-B (Fig. 2). No significant difference in hematological parameters, enzymes (ALT and GGT), and ALB protein were detected among groups (Table 1). The degree of inflammation in the wounded skin (epidermis, dermis, and fibrous connective tissue) varied from moderate to severe. However, no significant difference was found among treatment groups (Fig. 3a to c).

**Table 1:** Effect of CAMP-A and CAMP-B at 4 × MIC on the hematological and blood biochemical parameters of the treated mice

Category	Reference		0.9% NaCl	CAMP-A (4 × MIC)	CAMP-B (4 × MIC)
	Range	Unit	Average ± STDEV	Average ± STDEV	Average ± STDEV
Hematology					
WBC	0.80–10.60	10^3^/μL	6.664 ± 1.140	7.174 ± 2.609	6.081 ± 1.161
RBC	6.50–11.50	10^6^/μL	9.350 ± 0.345	9.503 ± 0.416	8.979 ± 1.026
HGB	11.0–16.5	g/dL	13.980 ± 0.349	14.271 ± 0.531	14.029 ± 1.005
HCT	35.0–55.0	%	44.200 ± 1.643	45.143 ± 1.952	43.143 ± 3.132
MCV	41.0–55.0	fL	47.200 ± 0.837	47.286 ± 0.756	48.286 ± 2.984
MCH	13.0–18.0	pg	15.000 ± 0.485	15.043 ± 0.282	15.729 ± 0.921
MCHC	30.0–36.0	g/dL	31.840 ± 1.163	31.700 ± 0.678	32.571 ± 0.854
Platelet Count	400–1600	10^3^/μL	1021.400 ± 155.307	937.714 ± 251.844	965.714 ± 122.545
Neutrophil	0.23–3.60	10^3^/μL	1.618 ± 0.437	1.433 ± 0.708	1.380 ± 0.621
Lymphocyte	0.60–8.90	10^3^/μL	4.568 ± 1.133	5.042 ± 2.140	4.041 ± 0.805
Monocyte	0.04–1.40	10^3^/μL	0.220 ± 0.189	0.191 ± 0.087	0.244 ± 0.168
Eosinophil	0.00–0.51	10^3^/μL	0.220 ± 0.068	0.459 ± 0.421	0.373 ± 0.22
Basophil	0.00–0.12	10^3^/μL	0.038 ± 0.019	0.063 ± 0.059	0.030 ± 0.017
Neutrophil	6.5–50.0	%	24.60% ± 0.08	21.71% ± 0.11	22.71% ± 0.10
Lymphocyte	40.0–92.0	%	68.00% ± 0.07	69.29% ± 0.09	66.71% ± 0.07
Monocyte	0.9–18.0	%	3.20% ± 0.03	2.71% ± 0.01	3.86% ± 0.02
Eosinophil	0.0–7.5	%	3.40% ± 0.01	5.57% ± 0.03	6.00% ± 0.03
Basophil	0.0–1.5	%	0.72% ± 0.004	0.73% ± 0.004	0.67% ± 0.004
Biochemistry					
ALT	27–195	U/L	46.400 ± 41.150	27.167 ± 8.704	75.500 ± 45.713
ALB	2.4–4.3	g/dL	2.960 ± 0.114	2.929 ± 0.160	2.840 ± 0.152
GGT	0–9	U/L	< 3 ± 0.000	< 3 ± 0.000	< 3 ± 0.000

*WBC* white blood cells, *RBC* red blood cells, *HGB* hemoglobin, *HCT* hematocrit, *MCV* mean cell volume, *MCH* mean cell hemoglobin, *MCHC* mean cell hemoglobin, *ALT* alanine transferase, *ALB* albumin, *GGT* gamma-glutamyltransferase

No tissue damage or significant inflammatory infiltration was observed in the skin prior to wound creation (Fig. 3d). The average scores of the inflammatory response based on infiltrating neutrophils and monocytes were similar among treatment groups (Fig. 3e). No CAMP-specific lesion or inflammation was observed in liver.

In the second toxicity study, CAMP-A at higher doses (5× and 50 × MIC) did not induce significant changes in body weight, behavior, and hair coat of the treated mice, which were similar to the observations during the first trial. In addition, CAMP-A treatments did not cause any changes in hematological parameters, but were associated with elevated ALT level (Table 2). The average ALT levels of mice treated with 5 × MIC CAMP-A and 50 × MIC CAMP-A were 148.3 ± 99.9 U/L and 103.2 ± 157.3 U/L, respectively, compared with 49.0 ± 28.7 U/L in the control group (Table 2). The elevated average ALT value was caused by the rise of ALT in a single mouse (312 U/L) in the 5 × MIC CAMP-A treatment group (14% of mice) and a single mouse (384 U/L) in the 50 × MIC CAMP-A treatment group (14% of mice). However, partial hemolysis was observed in both samples which might have skewed the ALT values. No significant difference in the inflammatory scores of the wounded skin was found among treatment groups. The inflammation score of mice treated with high doses of CAMP-A was comparable to that of the mice treated with low dose of CAMP-A. A few eosinophils and binucleated cells were observed in the liver of a mouse in the 50 × MIC CAMP-A group, which, however, were also observed in the livers of 2 mice in NaCl and polysporin-treated groups.

**Table 2:** Effect of CAMP-A at 5 × MIC and 50 × MIC on the hematological and blood biochemical parameters of the treated mice

Category	Reference		0.9% NaCl	CAMP-A (5 × MIC)	CAMP-A (50 × MIC)
	Range	Unit	Average ± STDEV	Average ± STDEV	Average ± STDEV
Hematology					
WBC	0.80–10.60	10^3^/μL	4.93 ± 1.27	5.86 ± 0.71	5.29 ± 0.78
RBC	6.50–11.50	10^6^/μL	8.96 ± 0.52	8.69 ± 0.31	8.74 ± 0.24
HGB	11.0–16.5	g/dL	14.84 ± 0.73	14.53 ± 0.25	14.50 ± 0.33
HCT	35.0–55.0	%	42.31 ± 2.11	41.25 ± 1.07	41.32 ± 1.24
MCV	41.0–55.0	fL	47.26 ± 0.49	47.53 ± 0.68	47.30 ± 0.14
MCH	13.0–18.0	pg	16.59 ± 0.28	16.70 ± 0.38	16.60 ± 0.13
MCHC	30.0–36.0	g/dL	35.09 ± 0.48	35.18 ± 0.53	35.15 ± 0.34
Platelet Count	400–1600	10^3^/μL	754.50 ± 157.60	805.75 ± 98.96	873.40 ± 62.92
Neutrophil	0.23–3.60	10^3^/μL	0.72 ± 0.30	0.92 ± 0.10	0.86 ± 0.28
Lymphocyte	0.60–8.90	10^3^/μL	3.81 ± 0.96	4.35 ± 0.63	3.97 ± 0.55
Monocyte	0.04–1.40	10^3^/μL	0.11 ± 0.05	0.20 ± 0.05	0.16 ± 0.04
Eosinophil	0.00–0.51	10^3^/μL	0.26 ± 0.15	0.36 ± 0.14	0.26 ± 0.06
Basophil	0.00–0.12	10^3^/μL	0.033 ± 0.020	0.038 ± 0.015	0.032 ± 0.020
Neutrophil	6.5–50.0	%	14.44% ± 0.06	15.73% ± 0.02	16.02% ± 0.04
Lymphocyte	40.0–92.0	%	77.61% ± 0.06	74.15% ± 0.04	75.25% ± 0.03
Monocyte	0.9–18.0	%	2.23% ± 0.01	3.35% ± 0.00	3.10% ± 0.01
Eosinophil	0.0–7.5	%	4.96% ± 0.02	6.13% ± 0.02	5.03% ± 0.01
Basophil	0.0–1.5	%	0.76% ± 0.00	0.65% ± 0.00	0.60% ± 0.00
Biochemistry					
ALT	27–195	U/L	49.000 ± 28.678	148.286 ± 99.876 ^a^	103.200 ± 157.301 ^b^
ALB	2.4–4.3	g/dL	3.186 ± 0.069	3.043 ± 0.127	3.160 ± 0.055
GGT	0–9	U/L	< 3 ± 0.000	< 3 ± 0.000	< 3 ± 0.000

*WBC* white blood cells, *RBC* red blood cells, *HGB* hemoglobin, *HCT* hematocrit, *MCV* mean cell volume, *MCH* mean cell hemoglobin, *MCHC* mean cell hemoglobin, *ALT* alanine transferase, *ALB* albumin, *GGT* gamma-glutamyltransferase

^a^ALT value was 312 U/L in one mouse in 5 × MIC CAMP-A treatment group

^b^ALT value was 384 U/L in one mouse in 50 × MIC CAMP-A treatment group

### Treatment efficacy of CAMP-A and CAMP-B

**Fig. 4:**
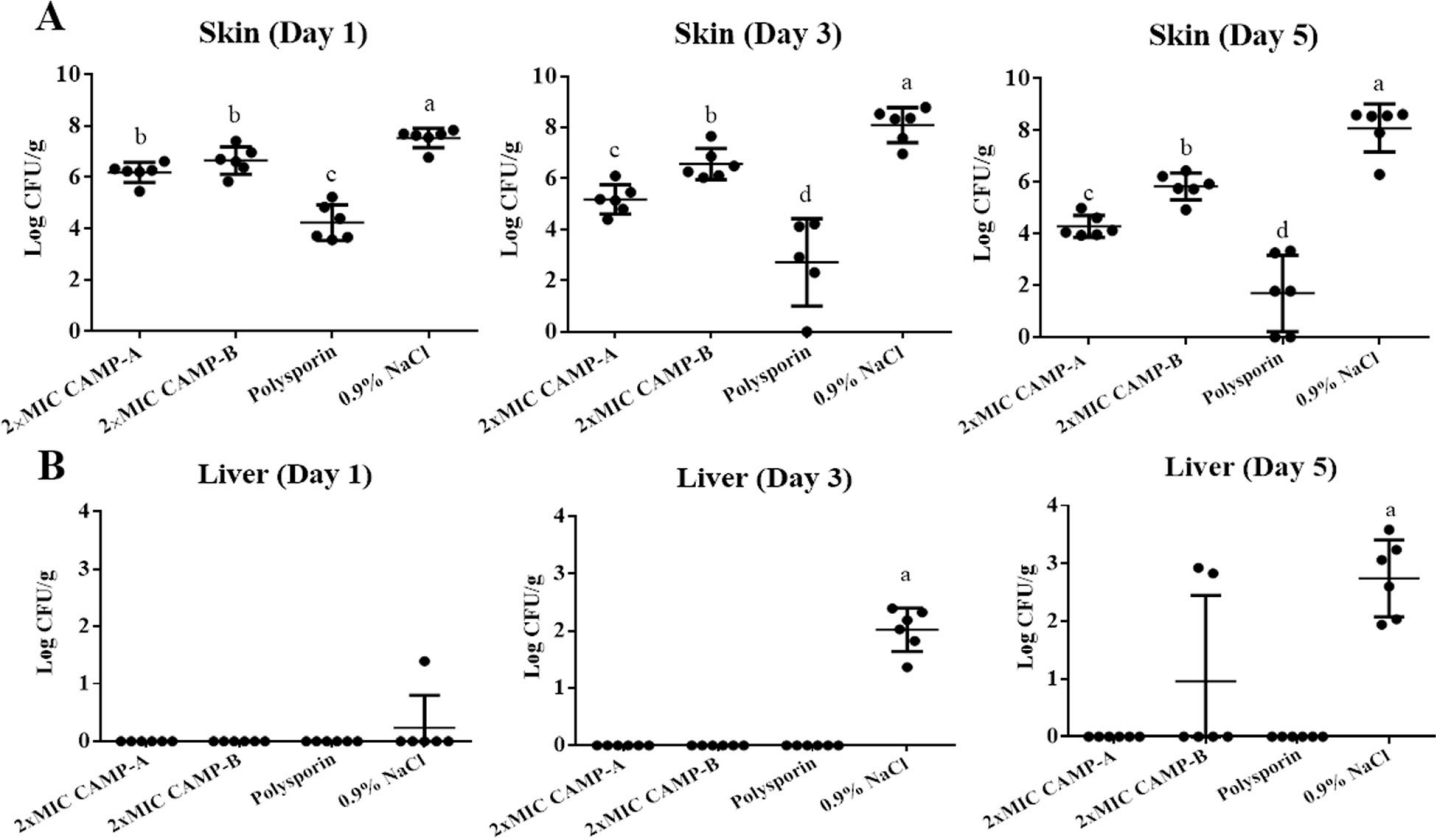
Efficacy of CAMPs at 2 × MIC in the clearance of wound bacterial infection and prevention of hepatic dissemination. The skin wound was inoculated with 2.5 × 10^6^ CFU of *P. aeruginosa* and treated daily with CAMP-A, CAMP-B, polysporin, or 0.9% NaCl. Bacterial counts (log_10_ CFU/g) were analyzed on day 1, day 3, and day 5. Data are presented as means ± SD (*n* = 6). **a** Skin bacterial burden. Different lowercase letters (a, b, c, and d) above bars show significant differences among treatment groups (One-way ANOVA followed by Duncan’s test; *p* < 0.05). Each letter represents a level of significant difference. **b** Liver bacterial burden. A lowercase letter (a) above bars indicates a significant difference between NaCl group and other treatment groups (One-way ANOVA followed by Duncan’s test; *p* < 0.05)

In the first trial, the wounds were treated with CAMP-A and CAMP-B at a relatively low dose (2x MIC). There was no significant difference in body weight and visual inspection scores among treatment groups (CAMP-A, CAMP-B, polysporin, and 0.9% NaCl) at any given day (Additional file 2: Figure S2). Significant differences in the clearance of wound infection and prevention of hepatic dissemination were observed among treatment groups. On day 1, the average bacterial counts in the wounds treated with CAMP-A, CAMP-B, and polysporin were 6.2 log_10_ CFU/g, 6.6 log_10_ CFU/g, and 4.2 log_10_ CFU/g, respectively, which were significantly lower than 7.53 log_10_ CFU/g in the mock-treatment group (*p* < 0.05, Fig. 4a). On day 3, the bacterial counts in wounds treated with CAMP-A, CAMP-B, and polysporin were reduced to 5.18, 6.6, and 2.7 log_10_ CFU/g, respectively, while the bacterial count in wounds treated with 0.9% NaCl was increased to 8.1 log_10_ CFU/g (*p* < 0.05, Fig. 4a). On day 5, the bacterial counts in CAMP-A, CAMP-B, and polysporin groups were further reduced to 4.3, 5.8, and 1.7 log_10_ CFU/g, respectively, and the bacterial count in 0.9% NaCl group remained at 8.1 CFU/g (*p* < 0.01, Fig. 4a). Treatment with CAMP-A and polysporin completely prevented *P. aeruginosa* infection in liver (Fig. 4b). Treatment with CAMP-B blocked hepatic dissemination in 4 of 6 mice (67%) and delayed the onset of liver infection by 2 to 4 days in 2 of 6 mice (33%) (Fig. 4b). In the mock-treatment group (0.9% NaCl), liver infection occurred in all 6 mice (Fig. 4b).

**Fig. 5:**
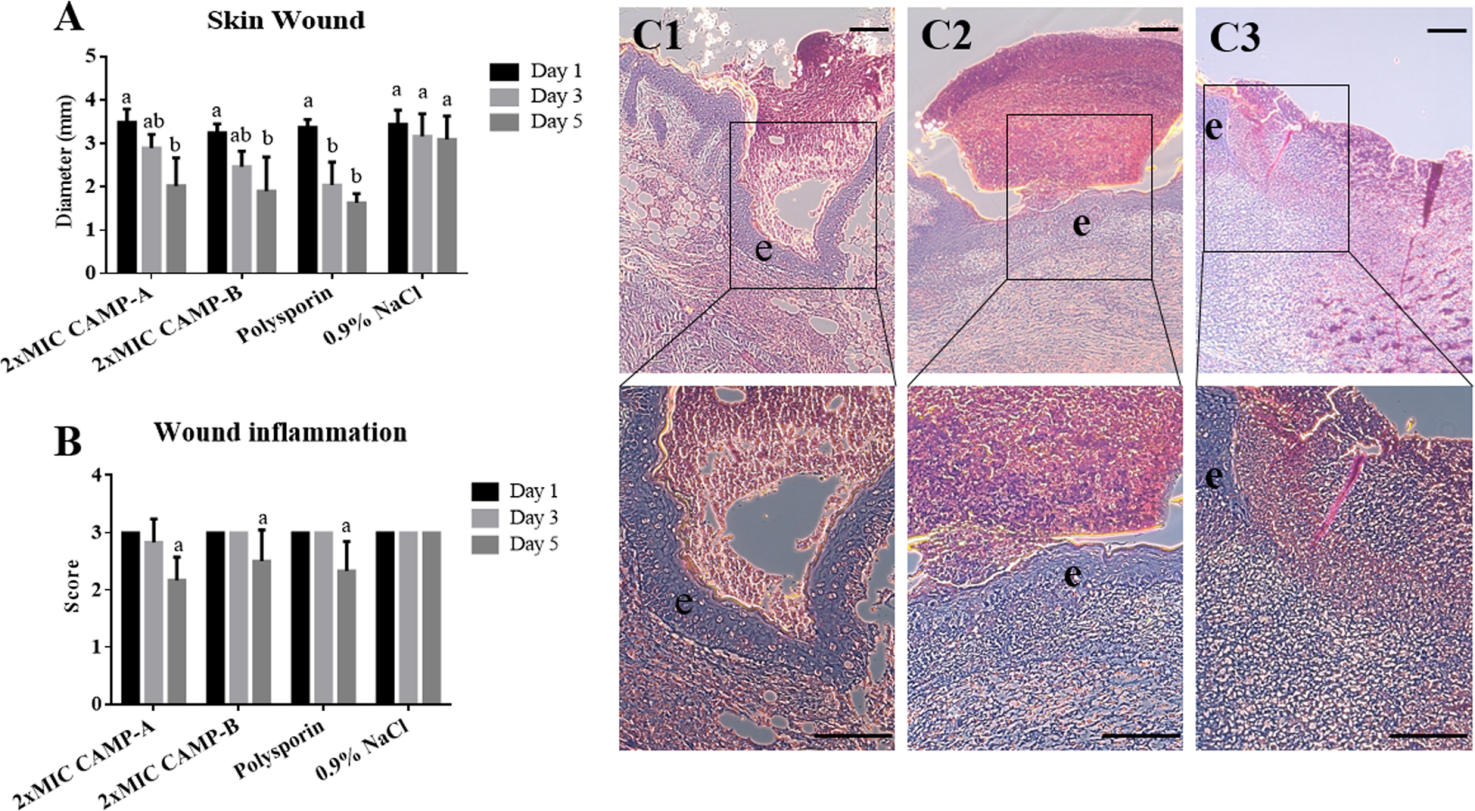
Efficacy of CAMPs at 2x MIC in promoting wound healing. Each wound was inoculated with 2.5 × 10^6^ CFU of *P. aeruginosa* and treated daily with CAMP-A, CAMP-B, polysporin, or 0.9% NaCl. **a** The diameter of the wounds. **b** Inflammatory scores of the infected skin on day 1, 3 and 5. Different lowercase letters (a, b) above bars indicate significant differences among treatment groups (One-way ANOVA followed by Duncan’s test; *p* < 0.05). Each letter represents a level of significant difference. **c** Epidermal cells proliferation induced by CAMP treatment (C1: CAMP-A, C2: CAMP-B, C3, 0.9% NaCl). e: epidermis. Bar = 100 μm. Data are presented as means ± SD (*n* = 6). Data are presented as means ± SD (*n* = 6)

In addition, treatment with CAMPs and polysporin promoted skin wound healing (Fig. 5a), although it did not change the overall severity of inflammation in the wounded skin (Fig. 5b). On day 1 post-treatment, no significant difference in the diameter of the open wound was observed among treatment groups (Fig. 5a). On day 3, the average diameters of the open wounds treated with CAMPs were significantly decreased, compared to those on day 1 as well as that in the mock-treatment group on day 3, but still larger than that in polysporin treatment group (Fig. 5a). On day 5, the diameters of the wounds treated with CAMPs were further reduced and comparable to that of the polysporin treatment group (Fig. 5a). Although no significant difference in wound inflammation was found among treatment groups (Fig. 5b), epidermal cell proliferation was observed in some CAMPs- and polysporin-treated mice on day 3 and day 5 (Fig. 5c), indicating the beginning of wound healing which correlated with wound closure.

**Fig. 6:**
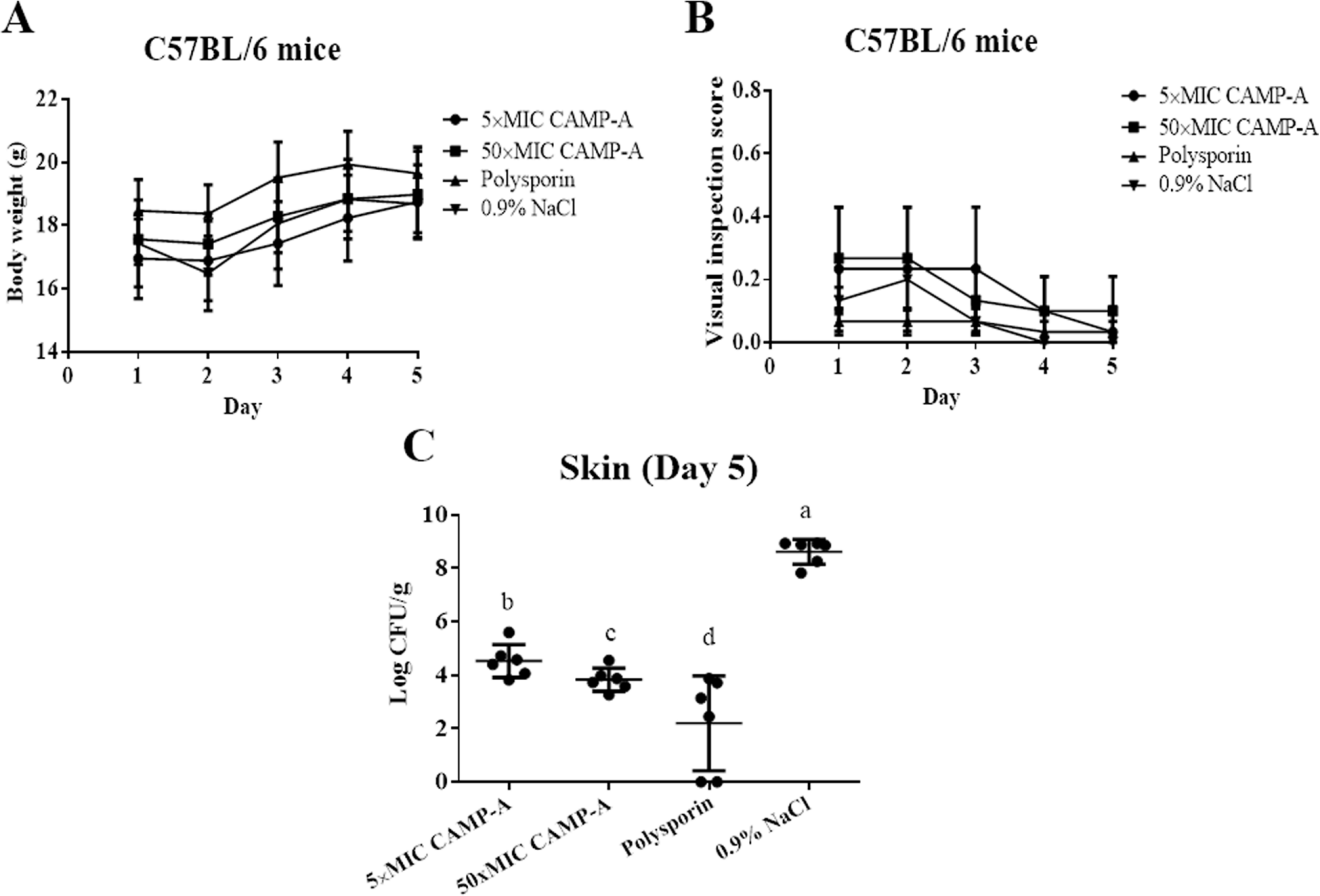
Efficacy CAMP-A at 5 × MIC and 50 × MIC in the clearance of wound bacterial infection. Each wound was inoculated with 2.5 × 10^6^ CFU of *P. aeruginosa* and treated daily with CAMP-A at 5 × MIC, CAMP-A at 50 × MIC, polysporin, or 0.9% NaCl. **a** Bodyweight of mice. **b** Visual inspection scores of physical activities and coat smoothness. **c** Skin wound bacterial burden (log_10_ CFU/g). Data are presented as means ± SD (*n* = 6). Different lowercase letters (a, b, c, and d) above bars indicate significant differences among different treatment groups (One-way ANOVA followed by Duncan’s test; *p* < 0.05). Each letter represents a level of significant difference

In the second trial, the wounds were treated with CAMP-A, a more promising peptide, at higher doses (5× and 50 × MIC). No significant differences in body weight (Fig. 6a) and visual inspection scores (Fig. 6b) were observed among treatment groups. On day 5, the bacterial counts in the wounded skin following treatment with 5 × MIC CAMP-A and 50 × MIC CAMP-A were 4.5 and 3.8 log_10_ CFU/g, respectively, which (Fig. 6c) were significantly lower than that achieved by administration of CAMP-A at 2 × MIC (5.18 log_10_ CFU/g). The wound bacterial counts for polysporin and NaCl treatment groups were 2.2 and 8.6 log_10_ CFU/g, respectively, which were in line with the outcome of the first trial. CAMP-A at both concentrations (5 × MIC and 50 × MIC) as well as polysporin completely prevented hepatic dissemination of *P. aeruginosa.*

## Discussion

In the previous study, an integrated strategy was used to design novel antimicrobial peptides possessing desirable characteristics as therapeutic agents. The data from in vitro studies indicated that CAMP-A and CAMP-B were broad-spectrum, potent antimicrobial peptides with resistance to cationic salts and proteases [17]. The strong in vitro antimicrobial activity of the peptides against *P. aeruginosa* multidrug-resistant clinical isolates laid the foundation for in vivo studies. In the present study, toxicity and anti-infectious efficacy the peptides were evaluated using a mouse skin wound infection model. It is known that skin, as a physical barrier, plays an important role in protecting the host from microbial invasion. When this barrier is compromised, such as in burn after injuries, opportunistic pathogens can establish local infection and then spread to systemic sites, if the wound is not properly treated [19, 20]. Among the known opportunistic pathogens, *P. aeruginosa* is a leading cause of opportunistic infection, especially in immunocompromised hosts [1]. *P. aeruginosa* can form biofilms with an association of gene modification, which makes it even much harder to kill [21], but the selected CAMPs are able to nonspecifically target on bacterial structure making them powerful [17].

To ensure the establishment of wound and liver infections, a relatively high dose of *P. aeruginosa* (2.5 × 10^6^ CFU) was inoculated into each wound and given 4 h prior to topical application of CAMPs. Bacterial cultural results indicated that *P. aeruginosa* proliferated rapidly in the surgical wound and spread to the liver of all experimental mice by 48 h.

In the present study, we evaluated the toxicity of CAMPs because toxicity could be a major hindrance in the development of antimicrobial peptides as therapeutic agents [22]. In addition to evaluating the physical behavior and body weight changes following CAMP treatment, we also analyzed the toxic effect of CAMPs on hematological and plasma biochemical parameters as well as plasma enzymes which are important indicators of illnesses or toxic effects of medical treatment [23]. The results indicated that daily application of 4 × MIC CAMP-A and CAMP-B on wounds for 7 days had no significant impact on the physical activities, body weight, hematological and blood biochemical profiles, and histology of wounded skin and livers of the treated mice. At higher doses (5× and 50 × MIC), elevation of alanine aminotransferase (ALT), an indicator of liver damage, was detected in 1 of 7 mice in each treatment group. However, partial hemolysis was observed in these samples. Although it is unclear whether the elevated ALT was caused by CAMP treatment or hemolysis [24], the percentage of affected mice was very small (14%).

Once the infection model was established and toxicity was assessed, the efficacy of CAMP-A and CAMP-B was evaluated by quantifying wound and liver bacterial burden, wound closure, inflammatory infiltration in the wounded skin and liver. Polysporin ointment containing polymyxin B (a lipopeptide antibiotic) and bacitracin (a mixture of cyclic peptides) was used as a positive control because it is commonly used to treat wound infections. Polysporin is not be recommended for long term use (> 5 days), due to potential nephrotoxicity and neurotoxicity of polymyxin B [25–27], despite that topical application would have limited systemic absorption. Out of precaution, the efficacy study was conducted using a 5-day regime. A recent study shows that frog skin-derived antimicrobial peptide promotes the migration of human HaCaT keratinocytes in an EGF receptor-dependent manner [28]. In the present study, we did not assess the wound healing property of the CAMPs because our initial in vitro characterization indicated that these peptides had no significant impact on the proliferation of JAWSII and CHO-K cells [17].

In the first trial, daily topical treatment with CAMP-A or CAMP-B at a low dose (2× MIC) reduced wound bacterial burden in a timely manner and facilitated wound closure in the course of 5-day treatment. CAMP-A completely prevented hepatic dissemination whereas CAMP-B prevented liver infection in 67% of mice and delayed systemic infection in 33% of mice. Inflammatory response in the wounded skin was seen throughout the course of experiments as expected due to surgical trauma [29]. Wound healing processes generally include an initial inflammatory response and subsequent proliferation and remodeling phase [30]. Data from the present study also indicated that CAMP-treatments promoted epithelial cell proliferation and wound closure without causing any side effects within the parameters of the study.

A second trial was carried out to further evaluate the efficacy of CAMP-A, a more potent antimicrobial peptide. Treatment with 50 × MIC CAMP-A for 5 consecutive days further reduced wound bacterial load, compared to treatment with 2 × MIC or 5 × MIC CAMP-A. However, complete clearance of *P. aeruginosa* from wounds was not achieved by CAMPs or polysporin, suggesting that longer treatment might be necessary as reported previously [31].

Compared to the commonly available polysporin ointment, CAMP-A and CAMP-B were less effective (*p* < 0.05) in reducing the bacterial burden in the infected wounds. Two factors might have contributed to this outcome. First, polysporin ointment contains both polymyxin B (10,000 units, 1000 μg /g) and bacitracin (500 units, 6750 μg/g) which damages bacterial cell wall and inhibits peptidoglycan synthesis. Second, the ointment ensures sustained interaction of drugs with bacteria in the wounded tissue whereas CAMP liquid was unable to adhere to the wounded skin. Further study is needed to explore ideal formulations.

## Conclusion

Topical application of CAMP-A and CAMP-B had no toxic effect on the experimental mice, except elevated ALT level in 14% of mice for which partial hemolysis occurred in the blood samples. The data collectively indicate that CAMPs significantly reduced wound bacterial load, promoted wound closure, and prevented hepatic dissemination. CAMP-A is a promising alternative to commonly used antibiotics to treat *P. aeruginosa* infection, although additional studies are needed to optimize delivery route, formation and treatment duration.

## Methods

### Peptides

The characteristics of two bactericidal peptides CAMP-A (LRRLKPLIRPWLRPLRRWWW) and CAMP-B (RRRWRKRRWW) were described previously [17]. As determined previously, the average of minimum inhibitory of concertation (MIC) of CAMP-A and CAMP-B were 15.27 and 52.36 μg/ml, respectively, for clinical isolates of multidrug-resistant *P. aeruginosa* [17]. All peptides were custom synthesized using the standard solid-phase 9-fluorenylmethoxycarbonyl (Fmoc) method and purified by reverse-phase high-performance liquid chromatography (RP-HPLC) (Lifetein, NJ) [14]. The purity of the synthetic CAMPs was greater than 98.5% as verified by liquid chromatography-mass spectrometry (LC-MS) (Lifetein, NJ).

### Animal source and care

The entire study, including the murine skin surgical wound infection model, was approved by the University of Missouri Institutional Animal Care and Use Committee (9047–2017) and Institutional Biosafety Committee (#14–20). The animal facility was accredited by Association for Assessment and Accreditation of Laboratory Animal Care International (AALAC) [32]. All procedures were performed in accordance with University of Missouri Institutional Animal Care and Use guidelines and the National Research Council Committee guidelines [33]. Specific-pathogen-free (SPF), female, 6-week-old, healthy C57BL/6 mice (body weight, 18 ± 2 g) were purchased from The Jackson Laboratory (Bar Harbor, MN). C57BL/6 mice were chosen due to their similar skin biological characteristics to that of humans [34] and C57BL/6 mice have been widely used to establish animal models for human diseases [35]. The number of mice used in each toxicity and efficacy studies was determined according to a power analysis with power (1-β error) 0.8 and α error probability of 0.05. Considering individual difference, total number of mice for each group was 5/0.9 = 6. One exception is the number of mice (4 per group) used to verify skin and systemic infection because the infection route and inoculating doses had been established previously [34]. The Institutional Animal Care and Use Committee also requires the use of reasonably the smallest number of animals in experiments. In total, 144 mice were used in the present study. The mice were randomly separated into different experiment or control groups. Prior to experiment, mice were raised one week in filter-top static cages for acclimation. During the course of experiments, mice were provided ad libitum with water and 5008 Formulab Diet (LabDiet, St. Louis, MO) and hosted individually in filter-top static cages in ABSL2 rooms under the following conditions: relative humidity between 30 and 70%, temperature between 20 and 26 °C, and a 12:12 h light: dark cycle. At the end of each experiment, mice were euthanized by exposure to gradually increasing concentration of carbon dioxide (10 to 30% per minute) according to University of Missouri Institutional Animal Care and Use guidelines.

### Creation of skin surgical wounds

The skin wound was created according to previously published methods with minor modifications [36]. Briefly, mice were weighed and anesthetized with an intraperitoneal injection of ketamine, 90 mg/kg (KetaVed, Vedco, St. Joseph, MO) and xylazine, 10 mg/kg (X-Ject E Injection, Henry Schein Animal Health, Dublin, OH). Mice were prepared for surgery by lubricating eyes, then clipping hair from the dorsal thorax through the neck region followed by aseptic scrub three times with povidone-iodine followed by 70% ethanol. After anesthetization, a 4 mm diameter full-thickness wound (both the epidermis and dermis) was cut using a sharp curved surgical scissor. A steam-sterilized black rubber washer with a pore of 6 mm inner diameter was carefully fixed around the skin wound using Cyanoacrylate adhesive (Zoetis, Parsippany, NJ). The rubber was further fixed with #4–0 nylon suture (Monosof™, Medtronic, Minneapolis, MN) through each quadrant of the washer and the surrounding skin, forming a splint to prevent healing by contraction. A piece of non-latex, hypoallergenic adhesive (Tegaderm Transparent Dressing, 3 M Animal Care Products, St. Paul, MN) was placed over the wound, completely covering while being trimmed to the edge of the rubber splint, to prevent peptide solution or saline leaking. The mice were given 0.05 mg/kg buprenorphine (Buprenex, Reckitt Benckiser Pharmaceuticals Inc., Richmond, VA) and anesthetic reversal, 1 mg/kg tolazoline (Tolazil, VET ONE, Boise, ID), both diluted in 0.9% NaCl (Hospira, Inc., Lake Forest, IL). Mice recovered in sterile filter-top covered cages.

### Verification of *P. aeruginosa* infection

*Pseudomonas aeruginosa* (ATCC 27853) was maintained in Luria-Bertani broth or agar (LB, BD *Difco*™) at 37 °C as described previously [15]. Briefly, the surgical wound was inoculated with approximately 2.5 × 10^6^ CFU of *P. aeruginosa* in sterile saline before covering the rubber washer. A total of 12 mice were used in this part of the study, including 3 groups for three time points and 4 mice per group. The mice in each group were sacrificed at 4, 24, or 48 h post-inoculation. Skin on the edge of wounds and liver were excised and homogenized. Serial dilutions of tissue homogenates were cultured on LB agar plates overnight at 37 °C and the colony-forming units (CFU) were recorded to determine bacterial load in the wound and systemic organs [37, 38].

### Evaluation of the toxic effect of CAMPs

The first toxicity study included 3 groups of mice, including 2 treatment groups and a control group (18 mice in total and 6 mice per group). Mice in the treatment groups received 50 μl of CAMP-A (4× MIC, 64 μg/ml) and CAMP-B (4× MIC, 128 μg/ml) via topical application. Control mice received a daily dose of 50 μl of sterile 0.9% NaCl for 7 days. The second toxicity trial also included 3 groups of mice (18 mice in total and 6 per group). Mice in two treatment groups received 50 μl of CAMP-A at 80 μg/ml (5 × MIC) and 800 μg/ml (50 × MIC) for 7 days, respectively. The control mice received a daily dose of 50 μl of sterile 0.9% NaCl for 7 days. The doses of CAMPs were chosen based on in vitro cytotoxicity study which indicated that CAMP-A at a concentration of > 4× MIC began to lyse murine red blood cells (less than 5%) [17]. Following topical applications, mice were observed daily for any signs of toxic effects. The toxicity of the peptides was determined by evaluating animal behavior, body weight, wound closure, hematological and biochemical parameters, histopathological changes in the wounded skin and liver. The following criteria were used to score changes in behavior, body weight, and smoothness of skin hair: no change in activity, body weight, and hair coat = no signs (0); quiet but active when touched, mildly scruffy hair coat, or up to 10% weight loss = minor signs (0.2); poor motility, moderately scruffy hair coat, or 10–20% of weight loss = mild signs (0.6); poor motility even under stimulation, very wiry hair coat, or > 20% of weight loss = manifest signs (0.8). According to the animal use protocol, veterinary care would have been provided to mice scored ≥0.8 and moribund animals were euthanized to avoid unnecessary suffering. At the end of study (day 7), blood was collected by cardiac puncture from each mouse into a Lithium Heparin tube (Greiner Bio-One) for hematological and biochemical analysis. Mice were sacrificed, and wounded skin and livers were collected for histological examinations.

### Evaluation of hematological and biochemical parameters

Hematological analysis was performed using the Sysmex XT2000i V Automated Hematology Analyzer (Sysmex America Inc., IL). The number of white blood cells (WBC), red blood cells (RBC), hemoglobin (HGB), hematocrit (HCT), platelet count (PLT), neutrophils (NEUT), lymphocytes (LYMP), monocytes (MONO), eosinophils (EOSI), basophils (BASO), and red cell indices: mean cell volume (MCV), mean cell hemoglobin (MCH), mean cell hemoglobin concentration (MCHC), and red cell distribution width (RDW), were counted. Blood biochemical profile analysis was carried out using the Olympus 400AUe Chemistry Analyzer (Olympus Corporation, PA). Alkaline aminotransferase (ALT), albumin (ALB), and gamma-glutamyltransferase (GGT) were used to assess liver functions.

### Examination of pathological changes

At necropsy, systemic organs were examined for gross lesions. Specimens, including the wounded skin and liver, were immediately fixed in 10% of formalin for 2 days. The fixed tissues were embedded in paraffin, sectioned at 5 μm thickness, and stained with hematoxylin and eosin [39]. Three sections of each tissue were evaluated and scored by a veterinary pathologist unaware of the treatment conditions. The following scale was used to score inflammation in skin and liver: 0 = none (no inflammation); 1 = minimal inflammation involving < 5% of tissue specimen; 2 = moderate with focally extensive areas of inflammation (5 to 25% of tissue, e.g. involving epidermis and dermis); 3 = moderate to severe with focally extensive areas of inflammation and fibrosis (> 25 to 50% of tissue, e.g. involving dermis, epidermis and subcutis).

### Determination of anti-infectious efficacy of CAMPs

The first treatment trial included 3 individual experiments for 3 treatment durations: 1 day, 3 days, and 5 days. Each experiment included 4 groups of mice (6 per group) for 4 different treatment options: CAMP-A (2 × MIC, 32 μg/ml), CAMP-B (2× MIC, 64 μg/ml), polysporin (Johnson & Johnson Consumer Inc., NJ), and 0.9% NaCl. A total of 72 mice were used in the first efficacy study (3 durations × 4 treatment groups × 6 mice per group = 72). The second trial consisted of a single experiment including 4 treatment groups: CAMP-A (5 × MIC, 80 μg/ml), CAMP-A (50× MIC, 800 μg/ml), polysporin, and 0.9% NaCl. A total of 24 mice were used in this part of the study (4 groups and 6 mice per group). The treatment in both trials continued for 5 days. Each gram of polysporin ointment contained 10, 000 units (1 mg) of polymyxin B and 500 units (6.75 mg) of bacitracin.

On the surgical day, each wound was inoculated with approximately 2.5 × 10^6^ CFU of *P. aeruginosa*. At 4 h post-inoculation, 50 μl of peptide, polysporin, or 0.9% NaCl was applied to the wound. The behavior, body weight, and wound closure of mice were recorded daily. When the treatment ended, mice were sacrificed. The skin of the wound area and liver were collected and processed separately for laboratory analysis. A portion of the tissue was homogenized for bacterial culture, and another portion was fixed in 10% formalin for histopathological evaluation. Treatment efficacy was determined based on wound healing, degree of inflammation, and bacterial burden in local and systemic organs.

### Statistical analysis

Differences among treatment groups were analyzed using the one-way analysis of variance (ANOVA) followed by Duncan’s test for multiple comparisons using software SPSS version 19.0 (IBM Corp., Armonk, NY). Differences at *p* < 0.05 level among different groups were considered statistically significant.

## Supplementary information

**Additional file 1: Figure S1.** Toxic effect of CAMPs on the body weight and physical activities of mice in the first toxicity study. Following administration of peptides, the body weight, physical activities, and hair coat were evaluated daily for 7 days. (A) Bodyweight and (B) visual inspection scores after treatment with 4 × MIC CAMP-A and CAMP-B in the first trial. (C) Bodyweight and visual inspection scores after treatment with 5× and 50× MIC CAMP-A in the second trial. Data are presented as means ± SD (*n* = 6). No significant difference was observed among treatment groups at any given time point in either trial.

**Additional file 2: Figure S2.** Effect of CAMPs on the body weight and visual inspection scores of mice treated with CAMPs at 2x MIC in the first efficacy trial. Four hours post-inoculation with 2.5 × 10^6^ CFU of *P. aeruginosa,* 50 μl of CAMP-A, CAMP-B, polysporin, or 0.9% NaCl were applied to the wound of each mice in appropriate experimental groups. Two separate experiments were conducted for 3-day and 5-day durations due to large numbers of mice involved. (A) Bodyweight and (B) visual inspection scores of physical activities and coat smoothness in a 3-day duration. (C) Bodyweight and (D) visual inspection of the activity and coat smoothness in a 5-day duration. Data are presented as means ± SD (*n* = 6).

## Data Availability

The datasets supporting the conclusions of this article are included in the article.

## Abbreviations

ALB: albumin
ALT: alkaline aminotransferase
ANOVA: One-way analysis of variance
AvBD: avian beta-defensin
CAMP: cationic antimicrobial peptide
CFU: colony-forming unit
ESI-MS: electrospray ionization mass spectrometry
FBS: fetal bovine serum
GGT: gamma glutamyl-transferase
HCT: hematocrit
HGB: hemoglobin
LB: Luria-Bertani
LPS: lipopolysaccharides
LTA: lipoteichoic acid
MCH: mean cell hemoglobin
MCHC: mean cell hemoglobin concentration
MCV: mean cell volume
MIC: minimum inhibitory concentration
PBS: phosphate-buffered saline
PLT: platelet count
RBC: red blood cells
RDW: red cell distribution width
SD: standard deviation
WBC: white blood cells
