# Supplementary figures and images for: Antimicrobial efficacy and toxicity of novel CAMPs against P. aeruginosa infection in a murine skin wound infection model
Source: BMC Microbiol. 2019 Dec 16;19:293. doi: 10.1186/s12866-019-1657-6 (PMC6915932; doi:10.1186/s12866-019-1657-6)

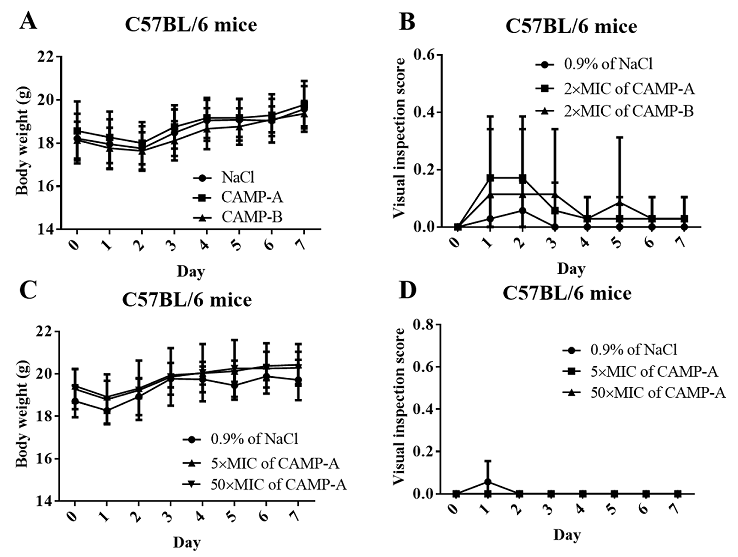

Supplement: Supplementary file 1 — Additional file 1: Figure S1. Toxic effect of CAMPs on the body weight and physical activities of mice in the first toxicity study. Following administration of peptides, the body weight, physical activities, and hair coat were evaluated daily for 7 days. (A) Bodyweight and (B) visual inspection scores after treatment with 4 × MIC CAMP-A and CAMP-B in the first trial. (C) Bodyweight and visual inspection scores after treatment with 5× and 50× MIC CAMP-A in the second trial. Data are presented as means ± SD (n = 6). No significant difference was observed among treatment groups at any given time point in either trial. [file 12866_2019_1657_MOESM1_ESM.tiff]

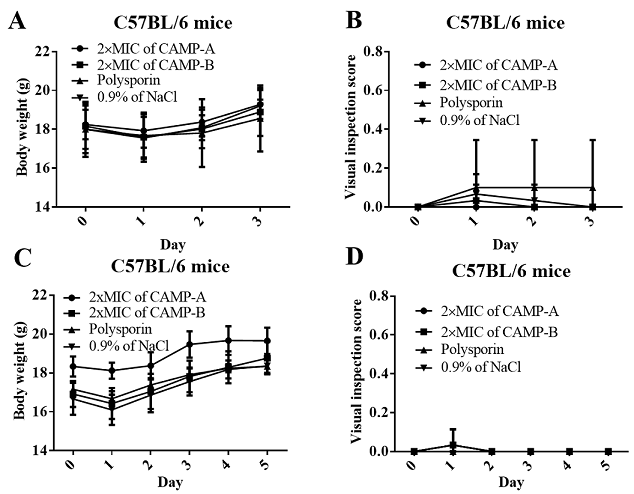

Supplement: Supplementary file 2 — Additional file 2: Figure S2. Effect of CAMPs on the body weight and visual inspection scores of mice treated with CAMPs at 2x MIC in the first efficacy trial. Four hours post-inoculation with 2.5 × 10^6^ CFU of P. aeruginosa, 50 μl of CAMP-A, CAMP-B, polysporin, or 0.9% NaCl were applied to the wound of each mice in appropriate experimental groups. Two separate experiments were conducted for 3-day and 5-day durations due to large numbers of mice involved. (A) Bodyweight and (B) visual inspection scores of physical activities and coat smoothness in a 3-day duration. (C) Bodyweight and (D) visual inspection of the activity and coat smoothness in a 5-day duration. Data are presented as means ± SD (n = 6). [file 12866_2019_1657_MOESM2_ESM.tiff]
